# Christmas Day at Guy's Hospital: A Wonderful Atmosphere of Unselfish Effort

**Published:** 1916-01-01

**Authors:** 


### CHRISTMAS DAY AT GUY'S HOSPITAL.

### A Wonderful Atmosphere of Unselfish Effort.

GUY'S HOSPITAL, for at least thirty years, has been a centre of life and brightness on Christmas Day, owing to the whole-hearted devotion of the members of the resident medical and nursing staff, a large number of old residents, and not a few of the visiting medical staff, who devote themselves to make this day memorable to all the patients in the wards by making it full of interest and blessedness for every one of them. The Governors also take a generous interest in the proceedings, and the Guy's Hospital Nigger Troupe has occupied so high a position amongst entertainers that it has sometimes made them in great request throughout the winter months.

WOUNDED OFFICERS IN COMPETITION.

The only noticeable difference between the celebrations in 1915 and previous years has been the fact that the decorations in some wards are slightly less elaborate, whilst in others, notably in the department set aside for wounded officers, a new departure has been taken by asking each patient to arrange for the decoration of his own cubicle, with results that are as novel as they are charming. One cubicle was occupied by an officer from the East, who brought with him an Eastern atmosphere, which he managed effectually and artistically to introduce into his scheme of decoration. In another case Japanese art appears to have been the inspiring force at work in the introduction of one of the most successful effects amongst many that were excellent. The effect in this case is deepened and improved by the addition of pictures on the walls. These give a homelike and attractive air of comfort which invites the passer-by to enter, sit down, and make himself or herself pleasant to the wounded officer. Some of the cubicles show a nautical tendency, being decorated throughout with flags—all flags, that is—that have a very pleasing effect when arranged with taste and artistic skill, which may be seen in some of them. Others have grotesque and wonderful designs in paper, including all sorts of colours, and representing many crude or arabesque or intermingled effects. There can be no doubt that the decorations in these cubicles mark a new departure in ward decoration, which it would be well to have photographed, so that the designs may be preserved for future use and extension.

AN OPPORTUNITY FOR MILLIONAIRES.

This department for wounded officers is remarkable from the fact that the floors are of rubber of different patterns and colours. To those accustomed to an ordinary hospital ward who find themselves on a rubber floor for the first time, there is a feeling of something strange, yet comforting about the department, which it is difficult to account for at first. When the novelty wears off a little, and the arrangements of the department, its furniture and plan, are mastered, those accustomed to hospital accommodation acquire the feeling that here is surely an almost ideal unit, in the sense that the complete stillness of everything must be an immense comfort to patients severely ill, and especially to those with head injuries or shock.

We have heard rich men complain that they would be grateful to know how they could wisely and helpfully expend a good round sum to make the less fortunate of the race more comfortable when ill than they are in existing conditions. Let any such person who reads this account make it their business to go to Guy's Hospital and study the rubber floor in the cubicles and the ward arrangements in the wounded officers' department. We make bold to think that if they do most if not all will agree that the rubber floors for hospital wards give them the very opportunity that they have been in search of. So we may look forward to the time when, in the paying departments of all our hospitals, rubber floors will be provided in a sufficient number of wards to enable all the severe cases to be placed there under the most favourable conditions to promote their rapid recovery and complete comfort. It is well, in selecting wards for rubber floors, to take care that they are not contiguous to some noisy street or thoroughfare, because the quietness due to the rubber aggravates the sounds from the streets and sometimes makes the patients suffer severely in consequence.

### The Boy Scout to the Rescue.

The Boy Scout has been reared upon books which exclude the word "impossible." The diversity of his occupations has never been few, and since the war he has become a sort of universal handyman. Hence it was natural to learn, in the course of our visit, that the Boy Scout had sometimes inspired a design, or, if he had not inspired it, that he had undertaken to go out and buy the necessary materials to make the cubicle of his employer second to none.

### The Large Wards and Out-patient Children.

Some of the large wards were as beautiful and attractive this year as ever, Bright and Job, to mention two only, being especially noticeable, as indeed were all the wards, for everywhere there was evidence of individual taste, and of an honest desire to do the best with the rather limited materials available this year, so as to brighten and cheer the occupants. A striking feature of Christmas Day at Guy's Hospital is the out-patients' department, where a tea is given to children, confined to old out-patients, who afterwards have a very varied but most enjoyable and merry entertainment, to their infinite delight. The assistant matron, Miss Hogge, and a number of assistants, worked hard and enthusiastically to secure this success.

### Well Done, Wardmaids.

An entire novelty was a choir organised entirely by the wardmaids of the hospital, who were dressed in various costumes, one of them representing Britannia, with a splendid brazen helmet that glittered in the light and was a joy indeed for the children. They sang extremely well, and lent most effective aid to the amusement of the children. Afterwards they found their way into some of the wards, and helped forward the entertainment there with gusto and success. We think it infinitely creditable to the wardmaids that they should have shown such admirable spirit, and everybody in the hospital was surprised and pleased that this should be so.

### Carol Singing at Daybreak.

Christmas commences at Guy's Hospital with some excellent carol singing early in the morning—a sort of serenade which, although it may disturb some sleeping beauties, is ever welcome and thoroughly appreciated. It is supposed, we believe, to be undertaken for the benefit of the sisters, but the audience is not limited to them but includes hundreds of others within hearing, who enjoy the excellent singing and start the day with brightened expectancy and cheerful hearts.

### The Spirit of Personal Service.

When one visits Guy's on these occasions, and proceeds from ward to ward, talking to the sisters, hearing of what has been done, seeing and enjoying the Nigger Troupe's efforts, conversing here and there with a patient and gathering his or her view of the entertainments and methods pursued, one gradually absorbs the spirit which underlies the whole of the proceedings. That is the spirit of personal service in the days of health in the cause of the sick, on the part of many of the youngest and healthiest members of our race, who gladly give up the whole day to try with all their might to make Christmas a real, living force, comfort, and blessedness to every one of the patients in Guy's Hospital, from the youngest to the oldest.

### The Sisters' Splendid Devotion.

It would be impossible to over-estimate the value and devotion of the work done by each sister. We have often felt that the poets have neglected a most fruitful and attractive field in omitting to study the administration of hospital wards, and the special work, responsibilities, and duties of the sister in charge of each. We have known many hundreds of sisters, some of whom have spent their lives in the work, who have about them an atmosphere of faith, and hope, and forbearance, with a courageous strength which are real forces not only in their work but in their association with others. All honour to these noble women, who typify some of the noblest and most attractive characteristics which have carried the fame of the best women of our race to the ends of the earth. On Christmas Day, and for some days before and after, they have to work like giantesses, yet the spirit within them is so real and absorbing that you seldom see one of them looking tired, and there is no earthly power, we are confident, that ever could make a sister on these occasions admit that she had any sense of fatigue, so long as there remained any single thing to be done to secure the well-being of the patients, the comfort of her visitors, and the glorious happiness of everybody who has the good fortune to spend some portion of Christmas Day in her ward.

We confess that on driving home after some hours spent in the wards of Guy's Hospital on the 25th ult., we felt twenty years younger, and experienced a light-hearted joyousness which helped us to promote the innocent brightness and pleasure of the Christmas dinner party.

